# Effects of physiological fatigue on basketball shooting performance: the moderating role of attentional focus

**DOI:** 10.3389/fpsyg.2025.1593182

**Published:** 2025-09-29

**Authors:** Yuanyuan Luo, Yingying Cao, Shuairan Li, Yan Shi, Peng Chen

**Affiliations:** ^1^Dazhou College of Traditional Chinese Medicine, Dazhou, China; ^2^School of Sports, Xi’an University, Xi’an, China; ^3^Sports Coaching College, Beijing Sport University, Beijing, China; ^4^School of Physical Education, Shaanxi Normal University, Xi’an, China; ^5^School of Physical Education, Ningxia Normal University, Guyuan, China

**Keywords:** fatigue, attentional focus, external focus, internal focus, basketball shooting, sports performance, physiological fatigue

## Abstract

**Objectives:**

This study examined the moderating effect of attentional focus on basketball shooting performance under varying fatigue levels.

**Methods:**

A 2 (Attentional Focus: External vs. Internal) × 3 (Fatigue Level: No Fatigue, Moderate Fatigue, Severe Fatigue) within-subject design was employed. Thirty male basketball players (mean age: 20.1 ± 0.3 years) completed 20 standardized free-throw attempts under each condition. Shooting accuracy was recorded, and repeated measures ANOVA was performed to analyze main and interaction effects.

**Results:**

Significant main effects were observed for attentional focus [F (1,29) = 8.15, *p* = 0.008] and fatigue level [F (2,58) = 26.32, *p* < 0.001] along with a significant interaction effect between attentional focus and fatigue level [F (2,58) = 4.27, *p* = 0.018]. Shooting accuracy under external focus (75.0%) was significantly higher than under internal focus (65.0%). Under severe fatigue, external focus resulted in a 15% higher shooting accuracy than internal focus, with this advantage increasing as fatigue levels intensified.

**Conclusion:**

Fatigue impairs basketball shooting performance; however, adopting an external attentional focus can mitigate its negative impact, particularly under severe fatigue conditions.

## 1 Introduction

Basketball games have phases of high-intensity anaerobic effort, such as fast breaks, defensive presses, or explosive shooting actions, alternating with low-to-moderate intensity aerobic phases or recovery periods. Under fatigue conditions, athletes must frequently execute complex technical movements, such as precise shooting (Aydemir and Cinar, 2019). Understanding how to maintain stable shooting performance under fatigue is a critical research issue in sports psychology and sports training.

Attentional focus instruction is a critical psychological factor influencing sports performance (Wulf, 2013). Research has shown that providing appropriate attentional focus guidance during practice or competition can significantly enhance both skill execution and learning efficiency (Neumann, 2019). Attentional focus is generally classified into internal focus and external focus. Internal focus directs attention toward one’s bodily movements, such as applying hand force or controlling limb movement details. In contrast, external focus is directed toward movement outcomes or environmental cues, such as tracking the basketball’s flight trajectory or focusing on the target hoop (Kristiansen et al., 2018). Research has consistently shown that external attentional focus instruction is generally more beneficial for sports performance and skill acquisition (Bull et al., 2023). In basketball free throws, focusing on the hoop or the ball’s flight path (external focus) significantly improves accuracy compared with concentrating on movement details such as wrist force (internal focus) (Sarhan, 2024). Studies have repeatedly demonstrated that athletes who adopt external attentional instructions achieve higher free-throw accuracy than those who use internal attentional instructions (Al-Abood et al., 2002; Zachry et al., 2005). The constrained action hypothesis primarily explains the mechanism underlying the effects of attentional focus. Wulf et al. (1998) proposed that directing attention toward the intended movement outcome (external focus) minimizes conscious control over motor execution, facilitating more automatic and efficient performance. In contrast, focusing on one’s bodily movements (internal focus) often leads to excessive self-monitoring, increasing cognitive interference and disrupting movement fluidity (Strick et al., 2023). In summary, adopting an external attentional focus enables athletes to perform motor skills more naturally and fluently, whereas an internal attentional focus may lead to excessive conscious control, disrupting movement fluidity. This theory has been validated across various sports, demonstrating that practicing with an external focus not only enhances movement accuracy and stability but is also associated with lower electromyographic (EMG) activity, suggesting that athletes can execute movements with more efficient neuromuscular control (Ghorbani et al., 2019).

Fatigue can be categorized into physical fatigue and mental fatigue. Physiological fatigue refers to the reduction in muscular capacity and endurance performance—such as increased time to complete a time-trial test or decreased pace during sustained activity—induced by intense or prolonged exercise. It is typically characterized by diminished force production, alterations in neuromuscular activation, and the depletion of key metabolic substrates (Wender et al., 2022). Whereas mental fatigue is associated with impaired attention and decision-making ability resulting from prolonged cognitive load or emotional stress (Cao et al., 2024). In basketball games, high-intensity competition often induces significant physical fatigue and increased cognitive load, particularly in the later stages of a match, which in turn impairs the execution of fine motor skills, such as shooting (Costa et al., 2022). Previous studies have extensively examined the effects of fatigue on basketball performance. On the one hand, mild to moderate physical fatigue has a relatively minor impact on shooting accuracy (Li et al., 2025), with research indicating that basketball players experience only a slight decline in accuracy under such conditions (Bourdas et al., 2024). On the other hand, as fatigue levels increase, motor skill performance deteriorates significantly (Sun et al., 2021). A systematic review and meta-analysis by Li et al. (2025) found that moderate levels of physical or mental fatigue have a limited impact on shooting accuracy. However, severe physical fatigue significantly reduces the success rate of high-difficulty shots, such as three-pointers. The authors further noted that under moderate and severe fatigue conditions, shooting accuracy declines significantly, with severe physical fatigue exerting the most pronounced negative effect on shooting precision. Additionally, mental fatigue negatively affects basketball performance by reducing free-throw accuracy and slowing decision-making reaction time (Filipas et al., 2021). In a mentally fatigued state, athletes struggle to maintain focus, exhibit slower reaction times, and experience increased error rates (Cao et al., 2022).

Both physical fatigue and mental fatigue impair an athlete’s ability to execute technical movements, albeit to varying degrees. While previous studies have separately examined the effects of attentional focus and fatigue on sports performance, research on their potential interaction mechanisms remains limited. Under high-fatigue conditions, increased physiological and cognitive load restricts attentional resources. In such scenarios, internal attentional focus may further add to the cognitive load, as monitoring one’s movement details becomes more demanding under fatigue. In contrast, external attentional focus may help redirect attention away from fatigue-induced discomfort and complex movement control toward a clear external target, thereby mitigating the negative impact of fatigue on performance. This suggests that under fatigue, external attentional focus instruction may be more effective than internal attentional focus instruction in maintaining or even enhancing performance. This assumption aligns with the constrained action hypothesis, which posits that when cognitive and physical resources are limited, reducing conscious attention to movement execution facilitates automaticity, thereby enhancing movement stability.

Building on the existing literature, this study aims to examine changes in basketball shooting performance from an “attentional focus × fatigue level” interaction perspective. The central research question is: Can different types of attentional focus mitigate the effects of varying fatigue levels on shooting performance? To address this question, the study proposes the following hypotheses: Main Effects: (1) Shooting accuracy will be significantly higher under external attentional focus than under internal attentional focus. (2) Shooting accuracy will progressively decline as fatigue levels increase. Interaction Effects: (1) A significant interaction between fatigue level and attentional focus is expected. (2) Under high-fatigue conditions, the performance advantage of external attentional focus over internal attentional focus will be more pronounced. (3) Under low or moderate-fatigue conditions, the difference in performance between external and internal attentional focus is expected to be smaller compared to the severe-fatigue condition, but still present. By experimentally testing these hypotheses, this study seeks to provide practical insights into the effective application of attentional focus guidance under fatigue conditions, thereby optimizing basketball shooting performance. The findings will contribute scientific evidence to support late-game performance strategies and fatigue-resistant training interventions.

## 2 Materials and methods

### 2.1 Participants

An *a priori* power analysis was conducted using G*Power 3.1.9.7. Based on a significance level of α = 0.05, statistical power (1–β) of 0.80, a medium effect size (f = 0.25), and an assumed within-subject correlation of 0.50, the analysis determined that a minimum sample size of 28 participants was required to detect significant effects in a 2 × 3 repeated-measures ANOVA (RM-ANOVA). To accommodate potential attrition or unusable data, 30 participants were ultimately recruited. The effect size estimate was informed by prior research examining the influence of physiological fatigue on basketball shooting performance (Brini et al., 2021; Li et al., 2025). This study recruited 30 male basketball players from Beijing Sport University (see Table 1). All participants were official members of either the university team or faculty teams, with an average age of 20.1 ± 0.3 years, an average height of 183.1 ± 6.1 cm, and an average training experience of 11.5 ± 1.4 years. Additionally, all players held the First-Class Athlete certification. Throughout the testing process, no participants experienced significant injuries or illnesses, and all completed the designated tasks. This study complied with the Declaration of Helsinki (Levine, 1999), and received approval from the Ethics Committee of Sports Science Experiments at Beijing Sport University (Ethical Approval No. 2024241H).

**TABLE 1:** Summary of participant characteristics.

Age (years)	Height (cm)	Weight (kg)	Body fat (%)	Years of training
20.1 ± 0.3	183.1 ± 6.1	85.3 ± 3.7	10.6 ± 3.5	11.5 ± 1.4

### 2.2 Experimental design

This study employed a 2 (Attentional Focus Type: External vs. Internal) × 3 (Fatigue Level: No Fatigue, Moderate Fatigue, Severe Fatigue) within-subject (repeated measures) design. The dependent variable was basketball shooting performance, assessed using free throws, with both successful free throws and shooting accuracy (%) recorded for each experimental condition. Attentional focus was designated as the control variable, with two instructional conditions: external attentional focus and internal attentional focus. Fatigue level served as the independent variable, categorized into no fatigue (control), moderate fatigue, and severe fatigue. To mitigate practice effects and order effects related to fatigue, a Latin square counterbalancing design was applied to determine the order of experimental conditions. Each participant completed testing under three distinct fatigue conditions across three separate sessions, with a minimum interval of 48 h between sessions to ensure sufficient recovery. To control for potential order effects, a 3 × 3 Latin square design was employed. Participants were randomly assigned to one of three fatigue condition sequences: (1) no fatigue → moderate fatigue → severe fatigue; (2) moderate fatigue → severe fatigue → no fatigue; or (3) severe fatigue → no fatigue → moderate fatigue. On each test day, participants performed two attentional focus conditions (internal vs. external) under the same fatigue level. The order of attentional conditions was randomized using a coin toss, with a 10 min interval between trials. To ensure that participants maintained the target fatigue level during this interval, a dual-control strategy was implemented that combined real-time monitoring and threshold-based physiological replenishment: Heart rate (HR) and rating of perceived exertion (RPE) were recorded every two minutes. If HR declined by more than 10 bpm or RPE fell below the predefined thresholds (≥ 13 for moderate fatigue; ≥ 17 for severe fatigue), participants immediately performed a brief replenishment protocol: one set of 20 m shuttle runs and vertical jumps for moderate fatigue, or two sets for severe fatigue. Recovery activities during the interval were restricted to slow walking or standing; participants were prohibited from sitting or stretching to prevent premature recovery. A random subsample of 12 participants underwent fingertip blood lactate testing at the 5 min mark of the interval. Lactate concentrations were maintained at 6.1 ± 0.8 mmol/L for moderate fatigue and 8.3 ± 0.9 mmol/L for severe fatigue—both significantly elevated compared to resting levels. Additionally, comparisons of pre- and post-interval HR and RPE revealed less than a 3% reduction, with no statistically significant differences (*p* > 0.10). This protocol ensured that the desired fatigue state was consistently maintained throughout the testing period. Furthermore, by counterbalancing the fatigue order and randomizing attentional focus conditions, the design minimized carryover effects and enhanced the internal validity of the experimental procedure.

### 2.3 Attentional focus

Under the external attentional focus condition, participants were instructed to focus on the basket hoop, emphasizing the shooting target and outcome (Zachry et al., 2005). Before executing a free throw, they received the following instruction: Fix your gaze on the center of the hoop, track the basketball’s flight trajectory, and visualize the ball swishing through the net. Conversely, under the internal attentional focus condition, participants were instructed to focus on their body movements, emphasizing movement execution (Novriansyah et al., 2019). The corresponding instruction was: Feel the force applied by your wrist and the motion of your fingers as you release the ball. Focus on your shooting hand position and follow-through. Before each shooting session, the experimenter provided verbal instructions. Additionally, during the shooting process, after every five shots, a brief verbal reminder was given to reinforce attentional focus. In the external focus condition, reminders included “Look at the hoop” or “Aim at the basket.” In the internal focus condition, reminders included “Wrist” or “Use force.” In both conditions, participants executed their shots at their natural shooting rhythm, without deliberately adjusting their shooting technique. To minimize unintended psychological influence, the experimenter maintained a neutral tone and refrained from providing any technical feedback.

### 2.4 Fatigue assessment

To precisely regulate and quantify different levels of physical fatigue, A standardized incremental exercise protocol was employed in this study, in which participants performed repeated high-intensity exercise bouts designed to deplete muscular energy reserves and reliably induce the target level of peripheral neuromuscular fatigue. Fatigue assessment incorporated both subjective and objective measures. The Borg Rating of Perceived Exertion (RPE) scale (Borg, 1982) was used to assess participants’ subjective perceived exertion. while the Firstbeat heart rate monitoring system (Firstbeat, Finland) was employed to collect heart rate (HR) and heart rate variability (HRV) data (Daanen et al., 2012; Parmar et al., 2025). Additionally, blood lactate levels were measured using a portable lactate analyzer (Lactate Scout 3, SensLab GmbH, Leipzig, Germany) to provide an objective evaluation of fatigue status (Billat et al., 1999; Tanner et al., 2010). Heart rate (HR) and heart rate variability (HRV) were recorded within 0–10 s after completion of the fatigue-induction protocol, during which participants maintained an upright and motionless posture for 30 s to obtain ultra-short-term HRV (lnRMSSD) values. Data were sampled at 1,000 Hz and analyzed using Firstbeat Sports software. HRV quality control adhered to the criterion of artifact correction ≤ 5% to ensure data integrity. Immediately following HRV measurement, the rating of perceived exertion (RPE) was verbally reported by participants and recorded in real time by the experimenter. Blood lactate concentration was measured using a portable Lactate Scout 3 analyzer (SensLab GmbH, Leipzig, Germany). Capillary blood was collected from the radial side of the index fingertip of the non-dominant hand, with the first drop discarded and the second drop applied to the test strip. To minimize transient fluctuations resulting from acute exercise cessation, blood lactate sampling was conducted 90–120 s after completion of the shooting test. For verification of fatigue maintenance, an additional blood lactate sample was collected at the 5th minute of the between-condition interval to confirm whether fatigue levels remained stable during testing. The fatigue manipulation check revealed significant differences in HR, RPE, lnRMSSD, and blood lactate across the three fatigue conditions (*p* < 0.001), with consistent trends observed (severe > moderate > no fatigue; lnRMSSD decreasing) (see Table 2).

**TABLE 2:** Heart rate (HR), rating of perceived exertion (RPE), heart rate variability (lnRMSSD), and blood lactate were statistically analyzed among three fatigue conditions.

Variable	No fatigue	Moderate fatigue	Severe fatigue	F value	*P*-value
HR (bpm)	85 ± 8	150 ± 9	175 ± 8	489.3	< 0.001
RPE (score)	7.0 ± 1.0	13.4 ± 1.0	17.2 ± 0.8	815.6	< 0.001
HRV (lnRMSSD)	3.9 ± 0.5	3.2 ± 0.4	2.7 ± 0.4	54.7	< 0.001
Blood lactate (mmol/L)	1.6 ± 0.4	5.3 ± 0.8	9.4 ± 1.2	308.7	< 0.001

In the no-fatigue condition, participants completed the shooting test in a fully rested state. To control for time-related variability across groups, participants in the control group engaged in a seated waiting task lasting approximately 10 min, mirroring the duration of procedures undertaken by the experimental groups. During this period, all vigorous physical activity and social interaction were strictly prohibited to maintain stable psychological and physiological states. A timer was used by research personnel to monitor interval durations between experimental phases. One-way ANOVA confirmed that the total experimental time did not significantly differ among the groups [F (2, 42) = 1.27, *p* = 0.29]. without any additional physical exertion. In the moderate-fatigue condition, participants underwent a moderate-intensity intermittent exercise protocol designed to induce moderate fatigue. This protocol included 10 sets of 20 m shuttle sprints, 30 consecutive jumps in place, and 20 rapid shooting drills, lasting approximately 5 min. Participants’ Rating of Perceived Exertion (RPE, 6–20 scale) was required to reach approximately 13 (“somewhat hard”), indicating a moderate level of fatigue. In the severe-fatigue condition, participants performed a higher-intensity, prolonged intermittent exercise protocol to induce significant fatigue. This protocol consisted of 20 sets of 20 m shuttle sprints, 20 consecutive basket-touch jumps, and 10 rapid dribble layups, lasting approximately 10 min with minimal rest intervals. Participants’ RPE score had to reach approximately 17 (“Severe fatigue,” characterized by a Borg Rating of Perceived Exertion (RPE) of 19–20, indicating a state approaching but not reaching complete exhaustion) to qualify as severe fatigue (Foster, 1998; Li et al., 2025). After each fatigue induction session, participants stood still and rested for 30 s before immediately beginning the free-throw shooting test, ensuring that the designated fatigue level was maintained throughout the task.

### 2.5 Experimental procedure

**FIGURE 1:**
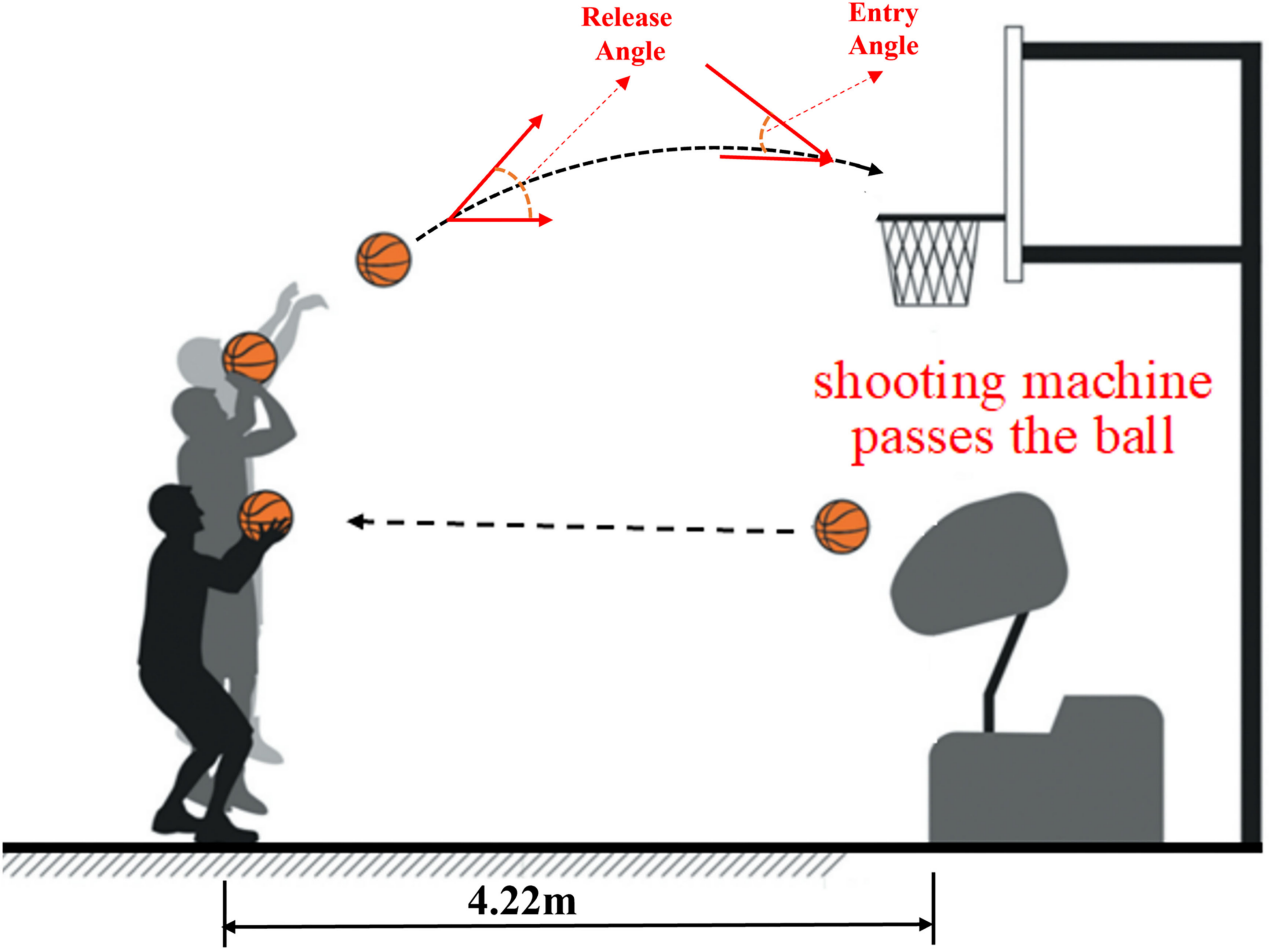
The illustration of jump shot testing (Reproduced from Li et al., 2021, licensed under CC BY-NC-ND 4.0).

The experiment was conducted in an indoor basketball gymnasium, with all participants tested between 2:00 p.m. and 5:00 p.m. to control for circadian rhythm effects on performance. Before the experiment, participants completed a 10 min standardized warm-up, including light jogging, stretching, and shooting practice, after which they were randomly assigned to one of three fatigue conditions. In the no-fatigue condition, participants proceeded directly to the shooting test, while in the moderate-fatigue and severe-fatigue conditions, they performed predefined fatigue induction exercises to reach the target fatigue level. Once the required fatigue state was achieved, participants immediately took position at the free-throw line, Under the external focus condition, participants received a standardized verbal cue: “Please focus on the basket.” To reinforce visual target localization, a high-contrast fluorescent orange marker (∼ 4 cm in diameter) was affixed to the center of the backboard. with the experimenter recording shooting accuracy without providing feedback. After completing the first set of 20 free throws, participants rested for 10 min. To ensure participants remained at the target fatigue state throughout this 10 min interval, we continuously monitored HR and RPE every 2 min and applied a threshold-triggered top-up (20 m shuttle runs + vertical jumps; 1 set for moderate fatigue, 2 sets for severe fatigue) whenever HR fell > 10 bpm from the immediate post-protocol value or RPE dropped below the preset thresholds (≥ 13 for moderate; ≥ 17 for severe); only slow walking/standing was permitted, and in a random subsample (*n* = 12) fingertip blood lactate at minute five remained elevated (moderate 6.1 ± 0.8 mmol⋅L^–1^; severe 8.3 ± 0.9 mmol⋅L^–1^), with pre- vs post-interval HR/RPE changes < 3% and non-significant (*p* > 0.10). Under fatigue conditions, they engaged in light activity (e.g., slow walking) to prevent full recovery and maintain an elevated heart rate, while during this period, the experimenter introduced the second attentional focus instruction. Under the internal focus condition, participants received a standardized verbal cue: “Please focus on the movement of your wrist during the shot.” To enhance bodily awareness, they were also instructed to perform a brief visualized movement rehearsal prior to shooting. This visualization-based approach has been validated in previous research as an effective method for promoting internal attentional focus (Wulf and Lewthwaite, 2016). Participants then completed another set of 20 free throws under the same fatigue condition but with the new attentional focus instruction. On each test day, participants completed trials under both attentional focus conditions, and if any fatigue conditions remained untested, the next session was scheduled at least 48 h later to ensure full recovery. Throughout the experiment, environmental variables and instructional procedures were strictly controlled to ensure that the only systematic variables between conditions were attentional focus instruction and fatigue manipulation. The free-throw shooting test procedure is illustrated in Figure 1.

### 2.6 Statistical methods

Shooting performance was assessed using shooting accuracy (%), calculated as the number of successful free throws out of 20 attempts, divided by 20, and then multiplied by 100%. Additionally, the raw number of successful shots was recorded for each experimental condition for statistical analysis. Each participant obtained one shooting accuracy score per experimental condition, resulting in six accuracy data points per participant (2 attentional focus conditions × 3 fatigue levels = 6 data points). Following data collection, statistical analyses were conducted using SPSS 26.0. First, means and standard deviations of shooting accuracy were calculated for each experimental condition as descriptive statistics. Prior to conducting the ANOVA, the normality of the dependent variable (shooting accuracy) was assessed using the Shapiro–Wilk test. Results indicated that the data met the assumption of normality (*p* > 0.05 for all conditions). Subsequently, a two-factor repeated-measures analysis of variance (ANOVA) was performed, with attentional focus and fatigue level as independent variables, to examine the main effects of attentional focus and fatigue level, as well as their interaction effect (Field, 2024). The significance level was set at α = 0.05 (Kirk, 2009). If a significant interaction effect was detected, further simple effects analysis and *post hoc* comparisons with Bonferroni correction were conducted to explore: (1) The effect of attentional focus under different fatigue conditions, the effect of fatigue under different attentional focus conditions (Rutherford, 2004). In addition, to validate the effectiveness of the fatigue induction protocol, participants’ immediate post-fatigue heart rate and RPE scores were recorded: Moderate-fatigue condition: Average heart rate ≈ 150 bpm, RPE ≈ 13.4, Severe-fatigue condition: Average heart rate ≈ 175 bpm, RPE ≈ 17.2 (Billat et al., 1999). Statistical analyses confirmed that heart rate and RPE scores differed significantly across fatigue conditions (severe fatigue > moderate fatigue > no fatigue, *p* < 0.001), indicating that the fatigue induction protocol successfully achieved the intended fatigue levels.

## 3 Research results

### 3.1 Comparison of HR, HRV, rating of RPE, and blood lactate levels across fatigue conditions

Significant main effects of fatigue condition were observed for heart rate (HR), rating of perceived exertion (RPE), heart rate variability (lnRMSSD), and blood lactate concentration (Table 2). One-way repeated-measures ANOVA revealed that mean HR values under the no-fatigue, moderate-fatigue, and severe-fatigue conditions were 85 ± 8, 150 ± 9, and 175 ± 8 bpm, respectively, F (2, 58) = 489.3, *p* < 0.001. *Post hoc* comparisons confirmed significant differences among all three conditions (severe > moderate > no fatigue, all *p* < 0.001). For RPE, the corresponding means were 7.0 ± 1.0, 13.4 ± 1.0, and 17.2 ± 0.8, respectively, F (2, 58) = 815.6, *p* < 0.001, showing a stepwise increase consistent with the HR pattern. Mean lnRMSSD values progressively decreased with fatigue severity, recorded as 3.9 ± 0.5 ms (no fatigue), 3.2 ± 0.4 ms (moderate fatigue), and 2.7 ± 0.4 ms (severe fatigue), F (2, 58) = 54.7, *p* < 0.001. Pairwise analyses indicated significant differences among all conditions (no fatigue > moderate > severe, all *p* < 0.001). Blood lactate concentrations also increased in a graded manner, with means of 1.6 ± 0.4, 5.3 ± 0.8, and 9.4 ± 1.2 mmol/L for the no-, moderate-, and severe-fatigue conditions, respectively, F (2, 58) = 308.7, *p* < 0.001. All pairwise comparisons were statistically significant (all *p* < 0.001). Collectively, these results demonstrate that the fatigue manipulation produced clearly distinguishable physiological and perceptual responses, thereby exhibiting strong discriminant validity and effectively inducing and maintaining distinct levels of peripheral neuromuscular fatigue.

### 3.2 Shooting accuracy across fatigue levels and attentional focus conditions

**FIGURE 2:**
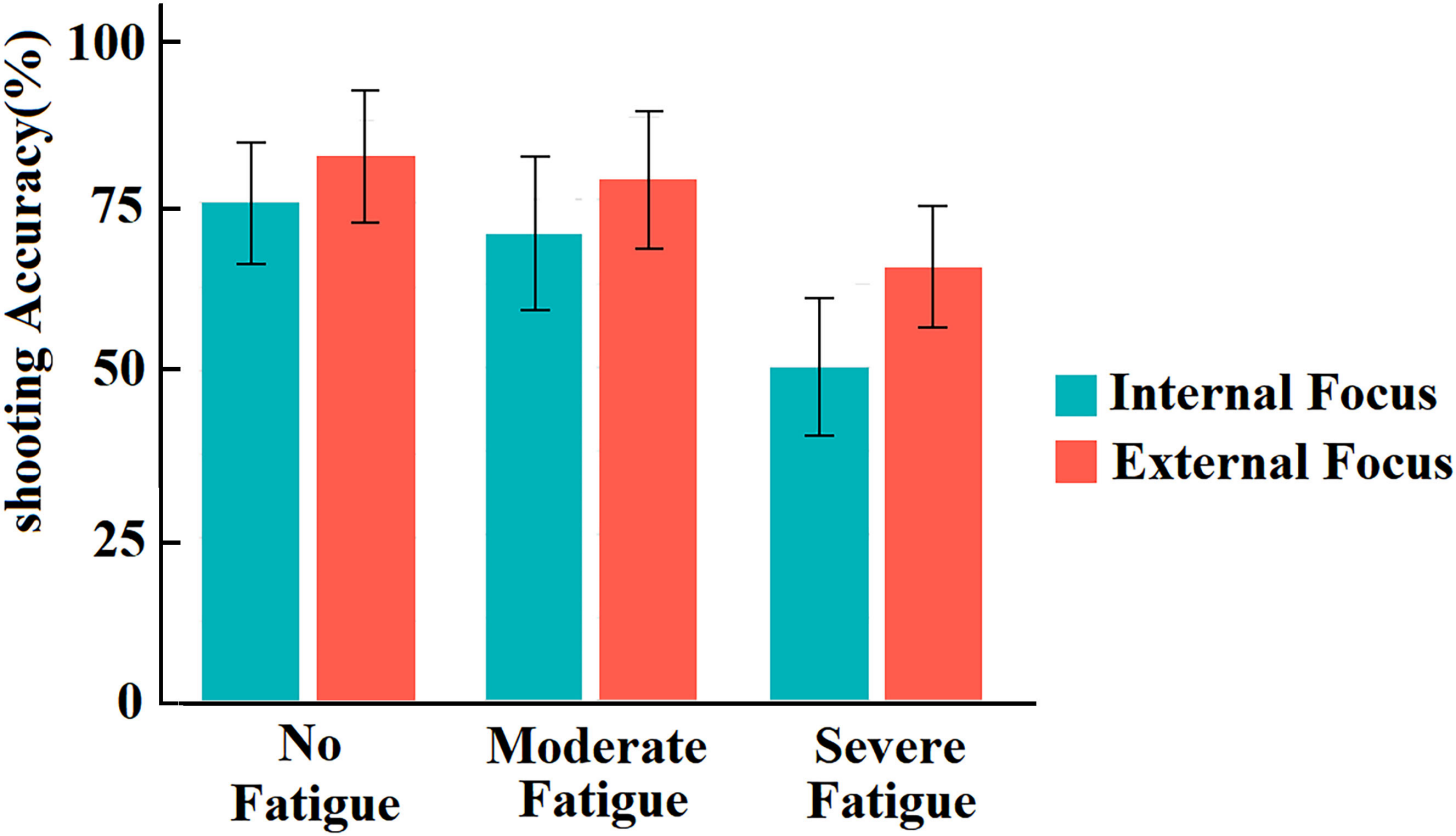
Shooting accuracy under different fatigue levels and attentional focus conditions.

Figure 2 illustrates the effects of attentional focus instructions on free-throw accuracy across varying fatigue levels. The results indicate that shooting accuracy progressively declines as fatigue levels increase; however, across all fatigue conditions, shooting accuracy remained consistently higher under external attentional focus compared to internal attentional focus. Specifically, in the no-fatigue condition, shooting accuracy was 82.0% ± 9.8% under external focus and 75.0% ± 9.0% under internal focus. In the moderate-fatigue condition, accuracy declined to 78.0% ± 10.2% (external) and 70.0% ± 11.5% (internal). Under severe fatigue, shooting accuracy further dropped to 65.0% ± 9.1% (external) and 50.0% ± 10.3% (internal). A notable finding is that in the no-fatigue condition, the difference in shooting accuracy between external and internal attentional focus was seven percentage points, whereas under severe fatigue, this gap expanded to 15 percentage points.

### 3.3 Effects of fatigue levels and attentional focus on shooting accuracy

As shown in Table 3, a repeated-measures ANOVA was conducted to examine the effects of fatigue level and attentional focus on shooting accuracy. The results indicated a significant main effect of attentional focus, with shooting accuracy being significantly higher under external attentional focus than under internal attentional focus [F (1, 29) = 8.15, *p* = 0.008], suggesting that attentional focus has a stable influence on shooting performance. A significant main effect of fatigue was also observed [F (2, 58) = 26.32, *p* < 0.001], with shooting accuracy progressively decreasing as fatigue levels increased. Additionally, a significant interaction effect between attentional focus and fatigue level [F (2, 58) = 4.27, *p* = 0.018] indicated that the effect of attentional focus on shooting accuracy varied across different fatigue conditions. Shooting accuracy in the external attentional focus condition was approximately 10% higher than in the internal attentional focus condition. Post hoc tests for the main effect of fatigue showed that the difference in shooting accuracy between the no-fatigue and moderate-fatigue conditions was not significant (*p* = 0.17), whereas shooting accuracy in the severe-fatigue condition was significantly lower than in both the no-fatigue (*p* < 0.001) and moderate-fatigue conditions (*p* < 0.001).

**TABLE 3:** Repeated-measures analysis of variance (ANOVA) results for attentional focus × fatigue level.

Source of variation	SS	df	MS	F	*P*	η^2^
Attentional focus (focus)	7200.05	1	720.050	8.15	0.008	0.22
Focus × within-subject error	2580.43	29	89.0000			
Fatigue	3560.32	2	1780.16	26.32	< 0.001	0.48
Fatigue × within-subject error	3920.78	58	67.6000			
Focus × fatigue	4630.40	2	231.700	4.270	0.018	0.13
Focus × fatigue × within-subject error	3156.66	58	54.4200			

This table reports the sum of squares (SS), degrees of freedom (df), mean square (MS), F-values, significance levels (*p*-values), and partial eta-squared (η^2^) for each effect term.

As shown in Table 4, Simple effects analysis revealed distinct patterns in how attentional focus instruction influenced shooting accuracy across different fatigue conditions. In the no-fatigue condition, although shooting accuracy was higher under external focus than internal focus, the difference was not statistically significant [*t*(29) = 1.73, *p* = 0.094, *d* = 0.74]. In the moderate-fatigue condition, the advantage of external focus further, showing a trend toward significance [*t*(29) = 1.86, *p* = 0.071, *d* = 0.74]. However, in the severe-fatigue condition, shooting accuracy under external focus was significantly higher than under internal focus [*t*(29) = 3.15, *p* = 0.003, *d* = 1.54], suggesting that external attentional focus can effectively mitigate the decline in shooting accuracy caused by fatigue.

**TABLE 4:** Simple effects of attentional focus on shooting accuracy at each fatigue level.

Focus type	Fatigue level	*t*(29)	*P*	Cohen’s *d*
External focus vs. internal focus	No fatigue	1.73	0.094	0.74
	Moderate fatigue	1.86	0.071	0.74
	Severe fatigue	3.15	0.003	1.54

As shown in Table 5, examining the effect of fatigue level under different attentional focus conditions, results showed that under external focus, there was no significant difference in shooting accuracy between the no-fatigue and moderate-fatigue conditions [*t*(29) = 1.20, *p* = 0.24, *d* = 0.40], but in the severe-fatigue condition, shooting accuracy was significantly lower than in both the no-fatigue [*t*(29) = 3.66, *p* < 0.001, *d* = 1.80] and moderate-fatigue conditions [*t*(29) = 3.40, *p* = 0.002, *d* = 1.35]. Under internal focus, shooting accuracy already showed a declining trend from no fatigue to moderate fatigue [*t*(29) = 1.81, *p* = 0.08, *d* = 0.48], and in the severe-fatigue condition, shooting accuracy was significantly lower than in both the no-fatigue [*t*(29) = 3.92, *p* < 0.001, *d* = 2.58] and moderate-fatigue conditions [*t*(29) = 3.66, *p* = 0.001, *d* = 1.83].

**TABLE 5:** Simple effects of fatigue level on shooting accuracy within each attentional focus condition.

Focus type	Fatigue level	*t*(29)	*P*	*Cohen’s d*
External focus	No fatigue vs. moderate fatigue	1.20	0.240	0.40
	No fatigue vs. severe fatigue	3.66	< 0.001	1.80
	Moderate fatigue vs. severe fatigue	3.40	0.002	1.35
Internal focus	No fatigue vs. moderate fatigue	1.81	0.080	0.48
	No fatigue vs. severe fatigue	3.92	< 0.001	2.58
	Moderate fatigue vs. severe fatigue	3.66	0.001	1.83

## 4 Discussion

This study employed a rigorous randomized controlled experiment to examine the effects of fatigue levels on basketball shooting performance under different attentional focus instructions. The results demonstrated that fatigue significantly impacted shooting accuracy, with performance declining progressively as fatigue levels increased, particularly in the severe fatigue condition, where shooting accuracy showed a notable decrease. Additionally, external attentional focus instruction was more effective than internal attentional focus in enhancing shooting accuracy, and this advantage persisted even under fatigue conditions. Furthermore, under severe fatigue, the performance advantage of external attentional focus became even more pronounced compared to internal attentional focus, indicating that the effectiveness of attentional focus is moderated by fatigue levels. The following discussion elaborates on these findings in greater detail.

Research has shown that basketball shooting, as a fine motor skill, requires precise body control, hand-eye coordination, and muscular strength. As physical fatigue increases, muscle strength output declines, movement precision decreases, and shooting stability and accuracy are compromised (Mulazimoglu et al., 2017). This study found that moderate fatigue did not significantly affect shooting accuracy, whereas severe fatigue caused a substantial decline in performance. This result aligns with the systematic review by Li et al. (2025), which concluded that mild to moderate fatigue has a limited effect on shooting accuracy, whereas severe fatigue significantly impairs shooting performance. One possible explanation is that moderate-intensity fatigue primarily affects fast movement capacity but has minimal immediate effects on shooting technique (Billat et al., 1999). Athletes may still rely on physical reserves or compensatory mechanisms (e.g., adjusting shooting force, leveraging technical experience) to maintain performance (Erčulj and Bračič, 2009). However, when fatigue accumulates beyond a critical threshold (e.g., RPE exceeding 17, approaching exhaustion), even repetitive free-throw motions become challenging to execute with consistent muscle output and focus, ultimately leading to a sharp decline in shooting accuracy (Foster, 1998). Additionally, this study observed that participants under internal attentional focus were more sensitive to fatigue, as shooting accuracy had already begun to decline in the moderate-fatigue condition. This suggests that fatigue not only affects physical capacity but may also impair technical execution through cognitive mechanisms. Under fatigue, attentional resources become limited. If an athlete must allocate attention to monitoring their movements (internal focus), they are more likely to experience attentional overload or diminished control, resulting in an earlier onset of performance decline (Mcguigan, 2017). In contrast, external attentional focus directs athletes’ attention toward an external target, thereby reducing awareness of fatigue-related discomfort and minimizing movement control interference. This delays the negative impact of fatigue on shooting accuracy (Wulf and Prinz, 2001). The findings showed that both external and internal focus conditions exhibited a non-significant decrease in shooting accuracy from no-fatigue to moderate-fatigue conditions, with the magnitude of the decrease being larger under internal focus (Wulf, 2013).

This study further confirmed the advantage of external attentional focus instruction in sports skill performance. Across all fatigue conditions, external attentional focus consistently resulted in higher free-throw accuracy compared to internal attentional focus. While this difference was relatively small and not statistically significant under no-fatigue and moderate-fatigue conditions, it became significantly larger under severe fatigue. This finding aligns with previous research (Chun et al., 2011; Singer et al., 1994; Wulf, 2013). Why Is External Attentional Focus More Beneficial for Sports Performance? (Wulf, 2013). Motor control theories provide a plausible explanation (Wulf and Prinz, 2001). When athletes over-focus on their movements, they tend to consciously control each detail, which interferes with the body’s natural coordination mechanisms, resulting in rigid and less fluid movements (Zachry et al., 2005). In contrast, directing attention toward the movement outcome (e.g., aiming at the target) enables athletes to rely on trained muscle memory and automatic control processes, facilitating more natural and efficient movement execution (Bell et al., 2009). Research by Zachry et al. (2005) further supports this perspective: when shooting under external focus conditions, electromyographic (EMG) activity in the shoulder and arm muscles was lower than under internal focus conditions, yet shooting accuracy was higher. This suggests that external focus allows athletes to achieve the same or even better movement outcomes with reduced muscle activation, thereby enhancing motor control efficiency (Lohse et al., 2010). Our experimental results reflected this effect, as shooting accuracy was consistently higher under external focus instruction. Furthermore, this advantage persisted even under fatigue conditions and became more pronounced in severe fatigue, reinforcing the robustness of external focus instruction. Regardless of whether an athlete is in a fully energized or fatigued state, excessive internal self-monitoring is detrimental to performance, while focusing on external targets effectively optimizes skill execution (Wulf, 2007).

A novel finding of this study is the significant interaction between attentional focus and fatigue level, specifically demonstrating that as fatigue increases, the advantage of external attentional focus over internal attentional focus becomes more pronounced, supporting our theoretical hypothesis. When athletes experience fatigue, both sensory feedback (e.g., muscle soreness, increased heart rate) and psychological stress (e.g., difficulty maintaining focus) intensify (Piccoli et al., 2018). If an athlete continues to focus on their movements, they must simultaneously process internal fatigue signals and movement control information, substantially increasing cognitive load, which may lead to movement distortion or attentional lapses (Beilock and Carr, 2001). In contrast, external attentional focus provides a clear and simple target, redirecting attention away from fatigue-induced discomfort and the complexities of movement control, resembling attentional shifting or simplification strategies that enable athletes to prioritize key movement elements even with limited cognitive resources, thereby maintaining more stable performance (Chen et al., 2023). Our experimental data support this hypothesis, as under high-fatigue conditions, participants guided by external focus still maintained a shooting accuracy of approximately 65%, whereas those under internal focus had an accuracy of only 50%, suggesting that external focus instruction mitigates fatigue-induced disruptions in shooting technique through mechanisms such as reducing fatigue-related attentional fluctuations (minimizing technical instability caused by reduced concentration), preventing excessive force compensation due to fatigue (helping to avoid movement distortion), and mitigating the negative psychological effects associated with fatigue awareness, thereby preserving an optimal performance state (Wulf, 2013). By contrast, internal focus instruction may exacerbate the negative effects of fatigue, as athletes not only experience increased muscle heaviness and weakness but also attempt to exert fine control over their shooting movements, leading to divided attentional resources and impaired motor execution, ultimately resulting in a sharp decline in shooting accuracy (Chen et al., 2023).

These research findings have significant theoretical implications, particularly in expanding the scope of attentional focus theory and understanding the interaction between cognitive and physiological factors. This study broadens the applicability of attentional focus theory, as previous research primarily examined its effects in non-fatigue or low-pressure environments. However, our findings demonstrate that under severe fatigue, a physiological stress condition, the advantages of external attentional focus not only persist but become even more pronounced. This suggests that the mechanism underlying external attentional focus remains robust despite changes in an individual’s physiological and psychological state and may even be more effective under increased stress. This conclusion aligns with previous research showing that external focus instruction enhances movement stability in high-pressure environments (Lohse et al., 2010; Zsidó et al., 2024). Furthermore, this study extends the concept of “pressure” to include “physical fatigue,” revealing that external attentional focus improves resistance to skill interference (Zhuravleva et al., 2023). These findings support the theory that external attentional focus enhances resistance to performance degradation under adverse conditions (Glotfelty, 2024), as athletes facing fatigue, anxiety, or other suboptimal conditions can maintain movement automatization, reducing the risk of skill breakdown or motor control failure (choking under pressure) (Zhou et al., 2025). Additionally, this study highlights the interaction between cognitive processes and physiological states, challenging traditional perspectives that classify fatigue as a purely physiological phenomenon and attentional focus as an isolated cognitive strategy (Martin et al., 2018). Our findings suggest that these two factors are not independent in influencing motor performance; instead, fatigue affects the brain’s allocation of attentional resources, while the effectiveness of attentional strategies is modulated by physiological state. This study provides a new theoretical perspective on how physiological and cognitive factors interact, offering deeper insight into athlete performance in the later stages of competition.

The findings of this study have significant practical implications for training and in-game coaching in basketball and other competitive sports. Coaches should consider integrating fatigue-based technical drills into training programs, such as conducting free-throw drills after conditioning exercises to simulate real-game scenarios in which athletes must execute shots under fatigue. During these sessions, coaches can guide athletes to adopt an external attentional focus (e.g., fixating on the hoop) to help them develop effective attentional strategies in fatigued states. In competitive settings, particularly during the final quarter or overtime, when athletes typically experience significant fatigue, coaches can incorporate attentional focus instructions during tactical timeouts. For instance, before free throws or critical shots, they can remind players: “Focus on the target, ignore movement details.” Brief external focus cues (e.g., “Fixate on the hoop,” “Aim at the center”) may be more effective than technical corrections, as the latter can increase cognitive load under fatigue and negatively impact execution (Fransen, 2024). Beyond in-game applications, attentional control should be a fundamental component of sports psychology training. Through systematic practice in shifting and maintaining optimal attentional focus under different conditions, athletes can develop the ability to use external focus during performance execution instinctively. Strengthening this mental skill not only reduces fatigue-induced errors but also enhances performance stability and decision-making in high-pressure situations (Montoro-Membila et al., 2025). In summary, this study highlights the importance of attentional focus strategies for both coaches and athletes. Specifically, external attentional focus serves as a simple yet effective technique for optimizing performance under fatigue, enabling athletes to execute critical plays more efficiently in high-stakes moments.

## 5 Research limitations and future directions

Despite its contributions, this study has certain limitations that warrant further exploration in future research. One key limitation is generalizability, as this study examined free-throw performance in male collegiate basketball players. Whether these findings apply to athletes of different competitive levels (e.g., professional players) or female athletes requires further validation, and caution should be exercised when generalizing the results (Reinebo et al., 2024). Additionally, basketball involves various shooting techniques, such as jump shots and three-pointers, which may be more dynamically affected by fatigue. Future research should expand task scope to investigate how fatigue and attentional focus influence different shooting techniques (Li et al., 2025). Another limitation is the study’s focus on physical fatigue, as it did not specifically examine psychological fatigue, such as that induced by prolonged cognitive tasks. Given that psychological fatigue can also impair shooting accuracy, future studies should incorporate psychological fatigue paradigms to explore its interaction with attentional focus, providing deeper insight into how different types of fatigue affect motor skill execution (Yoo et al., 2024). Additionally, as noted by Caterini et al. (1993), an internal focus of attention may be associated with distinct autonomic nervous system responses, such as decreased heart rate and elevated heart rate variability (HRV) during the preparatory phase. These physiological changes may affect the stability and precision of motor output, potentially compromising shooting accuracy. Future research is encouraged to incorporate such physiological indices into the experimental design of attentional focus studies—not only as manipulation checks, but also as physiological markers to elucidate the underlying mechanisms by which attentional focus modulates motor control under fatigue. Moreover, the present study did not implement a specific non-fatiguing task for the control group. While seated rest was employed to match the duration of procedures across conditions, this approach may have introduced structural inconsistencies, thereby limiting internal validity. Future studies are advised to adopt structurally equivalent but non-fatiguing physical tasks in the control condition to better control for non-specific confounders and enhance the methodological rigor of attentional focus interventions.

Additionally, while this study assessed the immediate effects of attentional focus on shooting accuracy, it did not examine long-term effects on skill acquisition and retention. Prior research suggests that, from a long-term perspective, external attentional focus enhances skill learning and retention, leading to improved performance in follow-up tests (Wulf, 2013). Future research could incorporate multiple training interventions to compare how different attentional focus strategies in fatigue conditions influence skill transfer and retention over time. Another important research direction is investigating the neurophysiological mechanisms of attentional focus under fatigue. Future studies could employ electroencephalography (EEG) or functional near-infrared spectroscopy (fNIRS) to monitor cortical activity during movement execution under different attentional focus conditions, providing neuroscientific insights into how external attentional focus mitigates fatigue-induced disruptions in motor control. These future investigations will deepen our understanding of the interaction between attentional focus and fatigue, providing a stronger scientific foundation for optimizing sports training and competition strategies. Moreover, although verbal instructions remain the most commonly used method for manipulating attentional focus, future research could improve the robustness and precision of such manipulations through several methodological enhancements: (1) incorporating visual cues—such as illuminated markers on the basketball hoop—to reinforce attentional direction; (2) employing eye-tracking technology to objectively verify gaze behavior and confirm attentional compliance (Vickers et al., 2019); and (3) integrating real-time biofeedback systems to guide and monitor attentional allocation dynamically during performance. In addition, future studies may consider repeating the “no-fatigue” condition twice within a single testing session or implementing a multi-day baseline assessment protocol. Additionally, the absence of baseline measurements may have allowed state-dependent factors on the testing day to introduce additional error, thereby limiting the precision of the effect size estimates. Accordingly, future studies should incorporate pre–post designs to more rigorously confirm and expand upon our findings.

## 6 Conclusion

This study demonstrated that both fatigue level and attentional focus type significantly influence basketball shooting performance. As physical fatigue intensifies, shooting accuracy declines substantially. However, adopting external attentional focus effectively enhances shooting accuracy, and this advantage persists even under fatigue conditions, becoming particularly pronounced in severe fatigue. In other words, external attentional focus helps mitigate the detrimental effects of fatigue on shooting performance. These findings support the constrained action hypothesis, which posits that reducing self-monitoring of movement and increasing focus on movement outcomes not only improves motor efficiency but also enhances athletes’ adaptability under adverse conditions. For basketball and other sports requiring precise technical execution despite late-game physical exhaustion, implementing external attentional focus strategies in training and competition can help athletes maintain optimal technical performance under fatigue. Importantly, the findings of this study should be understood as differences observed across fatigue levels, rather than as causal changes within the same testing session.

## Data Availability

The raw data supporting the conclusions of this article will be made available by the authors, without undue reservation.
